# Supplementary figures and images for: Service humanoid robotics: a novel interactive system based on bionic-companionship framework
Source: PeerJ Comput Sci. 2021 Aug 13;7:e674. doi: 10.7717/peerj-cs.674 (PMC8371998; doi:10.7717/peerj-cs.674)

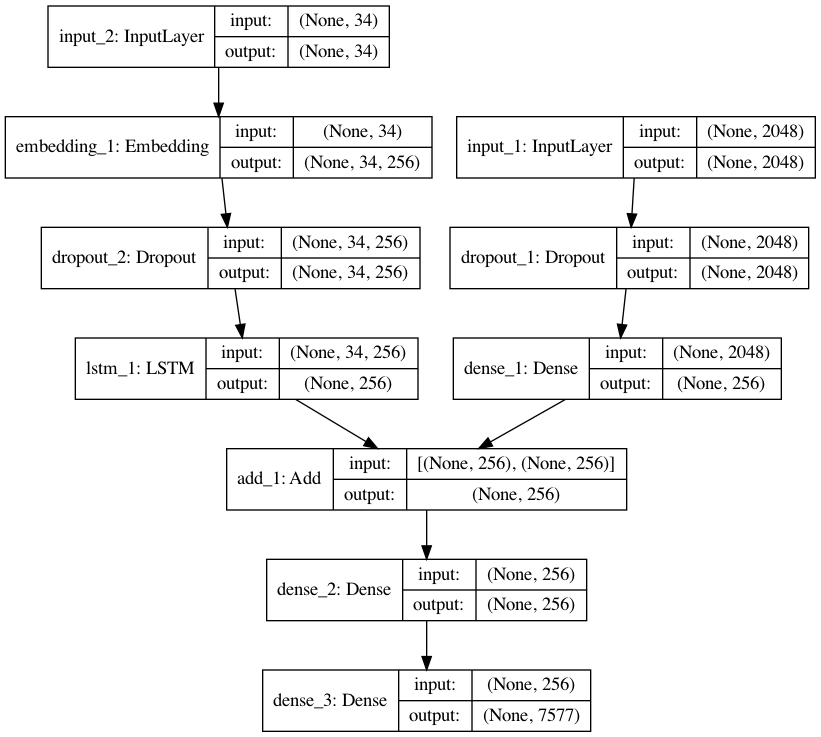

Supplement: Supplemental Information 1 [file peerj-cs-07-674-s001.zip › ImageCaptioning for Robot System/model.png]
